# Segment-Based SLAM Registration Optimization Algorithm Combining NDT and PL-ICP

**DOI:** 10.3390/s25237175

**Published:** 2025-11-24

**Authors:** Yi Zhang, Xiao Wang, Xiuqin Lyu, Liang Zhang, Weiwei Song, Rui Zhang

**Affiliations:** 1 School of Geodesy and Geomatics, Wuhan University, Wuhan 430079, China; yzhang@sgg.whu.edu.cn (Y.Z.); 15071462357@163.com (L.Z.); 2 School of Resource and Environmental Science, Wuhan University, Wuhan 430079, China; 3 GNSS Research Center, Wuhan University, Wuhan 430079, China; sww@whu.edu.cn; 4 Key Laboratory of Luojia of Hubei Province, Wuhan University, Wuhan 430079, China; zhangrui0221@whu.edu.cn

**Keywords:** solid-state LiDAR, SLAM, registration

## Abstract

With the continuous advancement of LiDAR technology, solid-state LiDAR, with its low cost and unique scanning mode, shows great potential in measurement applications. However, in large-scale environments, the SLAM algorithm LOAM-Livox for solid-state LiDAR often accumulates registration errors, limiting its applicability. To address this, we propose a segment-based SLAM registration optimization algorithm that combines Normal Distributions Transform (NDT) and Point-to-Line Iterative Closest Point (PL-ICP). This algorithm divides the entire data processing into segments, performs SLAM independently on each segment, and registers overlapping areas between adjacent segments to minimize error accumulation. Experiments on both public and self-collected datasets demonstrate that the proposed NDT + PL-ICP optimization algorithm significantly improves the accuracy of mobile mapping with solid-state LiDAR. This approach effectively resolves the error accumulation issue in SLAM, confirming its effectiveness and practicality in real-world applications.

## 1. Introduction

LiDAR technology is a widely used measurement technique in the field of surveying, with immense potential for development across traditional surveying, digital city construction, cultural heritage preservation, autonomous driving, and natural resource investigation and monitoring. Solid-state LiDAR, while having a smaller scanning angle, offers benefits such as compact size, low cost, and high scanning frequency, making it a popular research focus in the field of laser technology in recent years.

LOAM [1] marks a significant milestone, providing an efficient and accurate solution for real-time odometry and mapping through point-to-plane and point-to-line registration. LeGO-LOAM [2] improved upon it by segmenting and clustering planar points to extract ground features and employing two-stage optimization to enhance pose estimation accuracy and efficiency. LIO-SAM [3] further advanced the field with a tightly coupled lidar-inertial odometry and mapping framework, improving system robustness and adaptability. Ye et al. [4] propose a tightly-coupled LiDAR-IMU odometry framework that jointly optimizes lidar and inertial residuals within a fixed-lag smoother, enabling high-rate, drift-reduced state estimation under aggressive kinematics. Complementary to this, Lin et al. [5] introduce a rotation-invariant 2D histogram descriptor for rapid loop detection, yielding a complete LOAM-compatible closure module that significantly suppresses long-term accumulated error. Mendes et al. [6] develop an ICP-based pose-graph SLAM system that constructs keyframe-based local maps and performs graph optimization upon loop closure, effectively reducing drift and enhancing global consistency in large-scale environments. Droeschel et al. [7] present a hierarchical continuous-time SLAM approach that refines local multiresolution surfel maps and interpolates sensor trajectories using cubic B-splines, achieving online map refinement and improved alignment accuracy. However, these methods are primarily suited for rotational lidar sensors.

To address the challenges of small FoV and irregular scanning models in solid-state LiDARs, LOAM-Livox [8] develops a robust real-time odometry and mapping solution for the Livox MID-40 LiDAR. By fusing LiDAR features with IMU data, FAST-LIO [9] achieves computationally efficient and accurate LiDAR-inertial odometry through a tightly coupled iterated extended Kalman filter. This work is most similar to the proposed method, though ours leverages both geometry and intensity information to address degeneracy more effectively. FAST-LIO2 [10] enhances LiDAR localization and mapping by directly registering raw points to a global map, bypassing feature extraction, and utilizing the ikd-Tree toolkit. Leveraging the dense point cloud, CamVox [11] exploits the non-repeating scanning model for automatic calibration between a camera and Livox LiDAR. To improve accuracy and efficiency, LiLi-OM [12] introduces a tightly coupled LiDAR-inertial SLAM system based on keyframe-based sliding window optimization.

**Figure 1:**
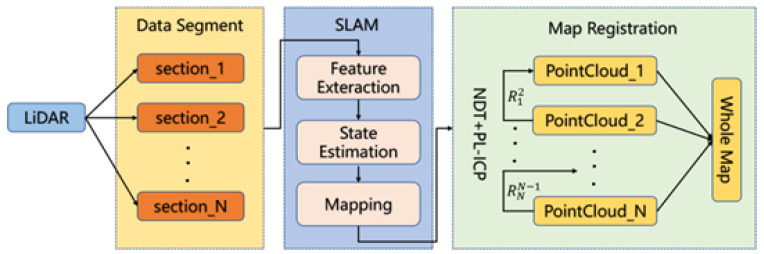
System pipeline. LiDAR data is segmented, individually processed with SLAM modules, and finally aligned using an NDT + PL-ICP fusion strategy for global map construction.

Due to narrow FoV, the accumulated errors over a large area can be significant, making the algorithm unsuitable for large-scale measurement tasks. This work proposes a segmentation-based registration optimization algorithm, combining NDT and PL-ICP, to divide the full point cloud into multiple segments for SLAM stitching. The algorithm then performs point cloud registration on overlapping regions, thereby enhancing the overall accuracy of point cloud stitching. The entire process is shown in Figure 1.

## 2. SLAM Algorithm Review

It primarily addresses the challenges of feature extraction in small FoV, robust outlier rejection, and dynamic object filtering. By directly matching each new frame with the global map, it provides odometry output, and the matching results are used to register the frame to the global map, achieving fast, robust, and high-precision LiDAR odometry and mapping.

### 2.1. Select Point and Extract Feature

**Figure 2:**
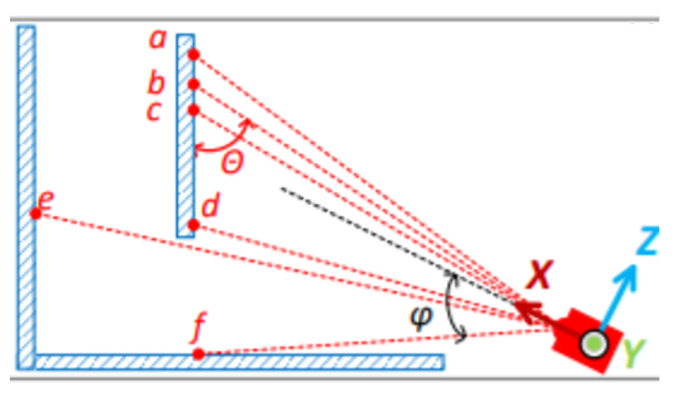
LiDAR scanning schematic diagram. The laser emits rays at discrete vertical angles Θ, capturing returns from structures (a–f) at varying depths and angles φ, relative to the local sensor coordinate system (X, Y, Z). a,b,c: normal returns; d,e: occluded points; f: high-incidence-angle.

Assume each laser point in the point cloud has coordinates *P* = [*x*, *y*, *z*], where the coordinates are defined in the local coordinate system of the current point cloud frame. As shown in Figure 2, the X-axis represents the laser emission direction, while the Z-axis points upward from the LiDAR sensor. Let *D* represent the distance of the laser point, which satisfies Equation (1):

(1) $$D\left(P\right)=\sqrt{x^{2}+y^{2}+z^{2}}$$

The laser deflection angle φ represents the angle between the laser beam and the X-axis, as shown in Equation (2):

(2) $$\emptyset \left(P\right)=\tan^{-1}\left(\sqrt{\frac{y^{2}+z^{2}}{x^{2}}}\right)$$

The intensity *I* of a point in the point cloud is given by Equation (3):

(3) $$I\left(P\right)=\frac{R}{D{\left(P\right)}^{2}}$$

where *R* denotes the reflectivity of the measured object. Typically, LiDAR point clouds contain intensity information. Low intensity in a point may indicate that the point is either far from the sensor or has a low object reflectivity.

Furthermore, although the reflectivity varies across different materials, it is important to note that low-intensity returns may indicate degraded ranging accuracy rather than just material properties. When the intensity is low, the laser echo signal is weaker, potentially leading to increased noise and uncertainty in the distance measurement.

The incidence angle θ, representing the angle between the laser beam and the plane around the point, is illustrated in Figure 1 with the calculation shown in Equation (4):

(4) $$\theta \left(P\right)=\cos^{-1}\left(\frac{\left(P_{a}-P_{c}\right)\cdot P_{b}}{∥P_{a}-P_{c}∥∥P_{b}∥}\right)$$

To enhance SLAM accuracy, it is necessary to evaluate each frame of point clouds during the SLAM process and remove the following points:

- **Points near the edge of the field of view**: For instance, in the Livox Mid-70 LiDAR, points with deflection angles exceeding 17 degrees tend to experience higher trajectory curvature in this region, which can lead to larger errors in feature extraction.
- **Points with excessively high or low intensity**: High intensity can cause saturation or distortion in the receiving circuitry, reducing range measurement accuracy. Conversely, low intensity results in a lower signal-to-noise ratio, also decreasing measurement accuracy.
- **Points with incidence angles close to π or 0**: As the laser beam diverges close to parallel with a plane, the laser footprint is significantly stretched, meaning the measured point coordinates represent an average over a large area rather than a single, precise point.
- **Occluded points**: Such points, shown in Figure 2 as e, can introduce errors during feature extraction.

Once point selection is complete, features are extracted from the chosen “good points.” Plane and edge features are identified by calculating the local smoothness for each point. To address the lower feature count caused by the narrow FOV, intensity values can be incorporated into the feature selection process. For instance, if a laser point’s intensity differs significantly from that of neighboring points, it can be considered an edge feature point. This approach is particularly useful for extracting features on surfaces with varying reflectivity, such as walls with doors and windows.

### 2.2. Pose Estimation and Optimization

Through the previous feature extraction process, edge and planar features have been identified for each frame of LiDAR point clouds. Next, the pose of each point cloud frame is iteratively optimized based on these extracted features. It is important to note that only the extracted edge and planar feature points of the current frame are used in the alignment, while the global point cloud is composed of the already aligned edge and planar feature points.

**Figure 3:**
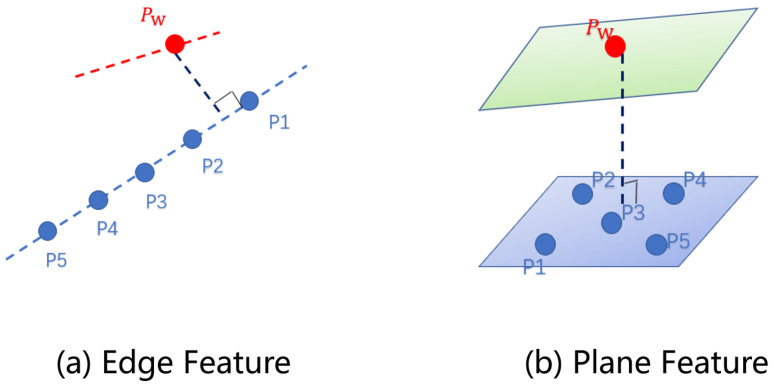
Feature constraints.

To estimate each frame’s point cloud pose, constraints must be constructed based on the extracted features shown in Figure 3.

**(i) Edge Feature Constraints**

Let $\varepsilon_{k}$ and $\varepsilon_{m}$ represent the edge features of the current frame and the global point cloud, respectively. Define $P_{l}$ as the set of laser points in $\varepsilon_{k}$. In this context, $P_{l}$ resides in the local coordinate frame of the current point cloud, which is the local LiDAR frame at the current timestamp, while all laser points in $\varepsilon_{m}$ are in the global coordinate frame, usually set as the coordinate system of the first point cloud frame. The pose of the current frame point cloud, defined as $\left(R_{k},T_{k}\right)$, is used to transform it from the local coordinate system to the global one, as shown in Equation (5):

(5) $$P_{w}=R_{k}P_{l}+T_{k}$$

where $P_{w}$ denotes the current frame’s transformed point cloud in the global frame. The final LiDAR scan point’s pose is used to represent the pose of the current frame, and the last laser point of the current frame aligns with the first point of the next frame. The initial values of $R_{k}$ and $T_{k}$ in each iteration are set as the pose of the previous frame.

Constraints are built using the Euclidean distance between point and edge features, following the principles of the PL-ICP algorithm, which enhances the robustness of SLAM over the traditional point-to-point ICP by using the Euclidean distance from points to lines in edge features. Each laser point in $P_{w}$ is iteratively matched with its five nearest neighbors $P_{i}$ (i=1,2,3,4,5) in $\varepsilon_{m}$, ensuring that these points form a line segment. This feature is verified through the covariance matrix of these five nearest points; if the largest eigenvalue exceeds three times the second largest, the points are considered collinear. The constraint *R* is the distance from each laser point in the current frame’s transformed edge feature to this line in the global cloud, as shown in Equation (6):

(6) $$R=\frac{∥\left(P_{w}-P_{1}\right)\times \left(P_{2}-P_{1}\right)∥}{∥P_{2}-P_{1}∥}$$

Similar to edge features, planar feature constraints rely on the Euclidean distance between planes. Let $\rho_{k}$ and $\rho_{m}$ represent the planar features of the current and global point clouds, respectively, with $P_{l}$ as the set of laser points in $\rho_{k}$. Here, $P_{l}$ resides in the local coordinate frame, while all points in $\rho_{m}$ are in the global frame. For each point in $P_{w}$, five nearest neighbors $P_{i}$ (i=1,2,3,4,5) are found in $\rho_{m}$ that form a plane. This plane is verified by evaluating the covariance matrix: if the smallest eigenvalue is less than one-third of the second smallest, these points are considered coplanar. *R* is defined as the Euclidean distance from each laser point in the transformed planar feature to the plane in the global cloud, as shown in Equation (7):

(7) $$R=\frac{{\left(P_{w}-P_{1}\right)}^{T}\left(\left(P_{3}-P_{5}\right)\times \left(P_{3}-P_{1}\right)\right)}{∥\left(P_{3}-P_{5}\right)\times \left(P_{3}-P_{1}\right)∥}$$

The constraint construction method for pose estimation calculates the Euclidean distances from line and plane features of the current frame’s laser points to their corresponding features in the global cloud.

### 2.3. Removal of Moving Objects

Moving objects can reduce SLAM accuracy, necessitating their exclusion. During constraint construction, edge feature constraints are formed by finding the nearest edge feature points and plane feature constraints are formed by finding the nearest planar points. During optimization, residuals are calculated for edge and plane constraints, and the top 20% with the largest residuals are excluded. This approach removes outliers and thus filters out moving objects during the optimization process.

## 3. Optimization Algorithm

The accumulated error in SLAM algorithms arises from incremental registration errors of each successive point cloud frame, leading to a cumulative drift in both the estimated LiDAR pose and actual point cloud positions, particularly over extended spatial and temporal ranges. However, when operating within shorter time intervals, the cumulative error of SLAM with a solid-state LiDAR remains relatively minor. Therefore, segmenting a long-distance, large-area scanning process into smaller, individual SLAM segments, and then performing registration across these segments, can significantly improve the overall accuracy of the motion measurement.

To enhance SLAM accuracy, inspired by ref [13], point clouds are processed in segments, with each segment undergoing independent SLAM registration and stitching. Adjacent segments have overlapping regions, and only these overlapping areas are used for registration to determine the transformation relationship (rotation matrix R and translation vector T) between them. Through registration of each pair of adjacent segments, we can derive the transformation relationship between them, thereby preventing the continuous accumulation of errors throughout the SLAM process.

Setting the first point cloud segment as the reference, let $P_{k}$ represent all laser points in the *k* segment, and let $R_{k}^{k-1}$ be the transformation matrix of the *k*-th segment relative to the previous segment. Here, $R_{k}^{k-1}$ is a 4×4 matrix comprising both rotation and translation components, satisfying Equation (8):

(8) $$P_{k-1}=R_{k}^{k-1}P_{k}$$

Thus, the transformation relationship of the *k* segment with respect to the reference point cloud can be expressed as shown in Equation (9). By determining the transformation relationship for each adjacent segment, all point clouds can be registered to the reference. The core of the segmented SLAM optimization algorithm lies in the registration of overlapping regions between two segments. Importantly, not all points in the two segments are used for registration; instead, only the overlapping region points are involved, as low overlap can lead to increased registration error. Therefore, the transformation relation for aligning each segment to the reference is as follows:

(9) $$P_{1}=R_{2}^{1}R_{3}^{2}\cdots R_{k}^{k-1}P_{k}$$

To achieve both speed and precision in this process, we adopt a hybrid registration strategy that combines Normal Distributions Transform (NDT) and Piecewise Linear Iterative Closest Point (PL-ICP). This combination leverages the strengths of both methods: NDT provides fast and robust coarse alignment by modeling the local spatial distribution of points within voxels, significantly reducing computational cost compared to global methods such as Generalized ICP (GICP). However, NDT’s resolution limits its fine alignment accuracy. To address this, PL-ICP introduces point-to-line constraints instead of traditional point-to-point ones, which are better suited for the sparse features of solid-state LiDAR and allow for more effective utilization of limited yet informative points for feature extraction and registration. By using NDT to estimate an initial transformation and PL-ICP to refine it, the algorithm achieves both high efficiency and registration precision—well-suited for large-scale, segmented SLAM optimization tasks.

### 3.1. Coarse Registration

The PL-ICP [14] algorithm has high demands for the initial pose estimation, thus requiring filtering and coarse registration of the point clouds to obtain the initial values for the registration process. To mitigate registration errors due to uneven point cloud distribution, voxel filtering is applied to downsample the vehicle’s point cloud data. This technique compensates for uneven distribution, reduces the overall number of points, enhances computational efficiency, and preserves essential point cloud features [15]. The primary principle of the voxel grid filter involves creating a 3D voxel grid within the spatial domain of the point cloud. Each voxel contains a reconstructed point, approximated by the centroid of all points within that voxel. The final filtered point cloud comprises the centroids of each 3D voxel grid [16]. The voxel grid size is determined based on the density of the point cloud and registration requirements, where larger voxels produce a sparser filtered point cloud. The calculation of the centroid within each voxel, containing m points, is as follows Equation (10):

(10) $$X_{j}=\frac{\sum_{i=1}^{m}x_{i}}{m},\;Y_{j}=\frac{\sum_{i=1}^{m}y_{i}}{m},\;Z_{j}=\frac{\sum_{i=1}^{m}z_{i}}{m}$$

Outliers in the point cloud may arise due to environmental factors or measurement noise, especially near edge regions. These isolated points are typically of low density and lack useful feature information, introducing significant error in registration and are thus removed. A statistical analysis method is applied to exclude these sparse outliers, where each point’s mean distance to its neighboring points is computed, yielding a Gaussian distribution. Points falling outside a defined threshold, based on standard deviation, are classified as outliers and removed. This method effectively eliminates a portion of point cloud noise.

In NDT [17], the point cloud is voxelized, creating a grid of cubic cells. The voxel size, or NDT resolution, must be set appropriately: too large a voxel size decreases registration accuracy, while a smaller size increases computation time. In each voxel, a dataset of scattered points is represented by a piecewise continuous probability density function, capturing both local feature orientation and smoothness, as well as voxel coordinates [11]. Gaussian distribution models the spatial probability distribution for each laser point within a voxel, as shown in Equation (11):

(11) $$P\left(\vec{x}\right)=\frac{1}{{\left(2\pi \right)}^{\frac{D}{2}}\sqrt{\left|\Sigma \right|}}\exp\left[-\frac{1}{2}{\left(\vec{x}-\vec{\mu }\right)}^{T}\Sigma^{-1}\left(\vec{x}-\vec{\mu }\right)\right]$$

where Σ and μ represent the covariance matrix and mean within each voxel, respectively, ensuring a probability density function with an integral of one. The calculations for Σ and μ are detailed in Equation (12):

(12) $$\mu =\frac{1}{m}\sum_{k=1}^{m}y_{k},\;\Sigma =\frac{1}{m}\sum_{k=1}^{m}\left(y_{k}-\mu \right){\left(y_{k}-\mu \right)}^{T}$$

The NDT algorithm follows these steps: (1) Set voxel size and compute probability distribution for each voxel. (2) Transform the point cloud according to the transformation matrix, computing the normal distribution for each transformed point. (3) Calculate a scoring metric S. (4) Optimize the score using the Hessian matrix method.

Through point cloud filtering and NDT coarse registration, reliable initial values for fine registration are obtained.

### 3.2. Fine Registration

**Figure 4:**
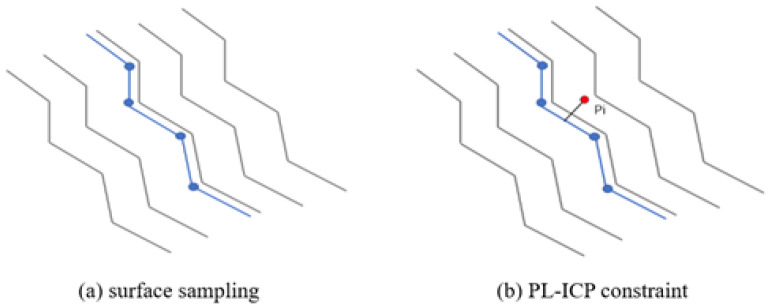
PL-ICP schematic. (**a**) Based on geometric features, blue points are extracted from structured environments. (**b**) Illustration of PL-ICP constraints, where each red point is registered by minimizing point-to-line and point-to-plane residuals.

Laser points are essentially discrete samples of real-world surfaces, allowing constraints to be constructed based on the distance from laser points to the actual surfaces. The PL-ICP algorithm simulates real surfaces using a piecewise linear approach, representing the distance from each laser point to the closest line segment formed by its two nearest points in the reference point cloud. This approach enables the PL-ICP error equation to more closely approximate the actual conditions, see Figure 4.

The main steps of the PL-ICP algorithm are as follows: Let the point cloud be registered be $P_{i}$, with the pose of the point cloud relative to the reference point cloud coordinate system represented as $\left(t_{k},\theta_{k}\right)$. Then, $P_{i}$ is transformed to the reference coordinate system as shown in Equation (13), where $p_{i}^{w}$ denotes the transformed point cloud:

(13) $$p_{i}^{w}≜p_{i}⊕q_{k}=R\left(\theta_{k}\right)p_{i}+t_{k}$$

For each laser point in $p_{i}^{w}$, the algorithm identifies the two nearest points in the reference point cloud. Here, a KD-tree indexing structure is employed to expedite this search. The indices $j_{1}^{i}$ and $j_{2}^{i}$ denote the two closest neighbors for the *i*-th point in $p_{i}^{w}$, and a tuple $C_{k}=\langle i,j_{1}^{i},j_{2}^{i}\rangle$ is constructed to represent this relationship.

A cost function is then established, as shown in Equation (14), where $n_{i}^{T}$ represents the normal vector of the line segment formed by the two nearest points. This cost function essentially computes the distance from each point in $p_{i}^{w}$ to the line connecting its two closest points in the reference cloud:

(14) $$J\left(q_{k+1},C_{k}\right)=\sum_{i}{\left(n_{i}^{T}\left[R\left(\theta_{k+1}\right)p_{i}+t_{k+1}-p_{j_{1}^{i}}\right]\right)}^{2}$$

By minimizing the function in Equation (14), the optimal pose for the point cloud to be registered can be iteratively computed. Since PL-ICP uses second-order optimization, it converges faster than the ICP algorithm. However, because PL-ICP is highly sensitive to initial values, the NDT algorithm is employed to estimate the initial pose. This completes the refinement of the segmented localization results in SLAM.

## 4. Experiment

### 4.1. Livox Open Data

The entire point cloud dataset is divided into multiple segments, with each segment consisting of around 1000 frames and a 40% overlap with the adjacent segment. Each segment is further divided into seven sub-segments. First, each individual segment undergoes standalone SLAM stitching to obtain a stitched point cloud for that segment. The overlapping sections of these point clouds are extracted for use in the NDT + PL-ICP registration process, which requires calculating six transformation matrices: $R_{1}^{2},R_{2}^{3},R_{3}^{4},R_{4}^{5},R_{5}^{6},R_{6}^{7}$. For instance, $R_{2}^{3}$ represents the transformation relationship between segments 2 and 3. Once these transformations are determined, the relative transformations to the first reference segment are computed using Equation (9), achieving the final stitching of the entire point cloud.

A segment length of approximately 1000 frames strikes a balance between computational efficiency and registration accuracy. Using fewer frames results in shorter durations and fewer overlapping point clouds, which reduces the accuracy of registration. Conversely, using too many frames introduces redundancy, significantly increasing computational cost. Similarly, a 40% overlap ratio was empirically determined to be optimal for reducing trajectory discontinuities, while ensuring a sufficient number of redundant points for robust inter-segment matching.

**Figure 5:**
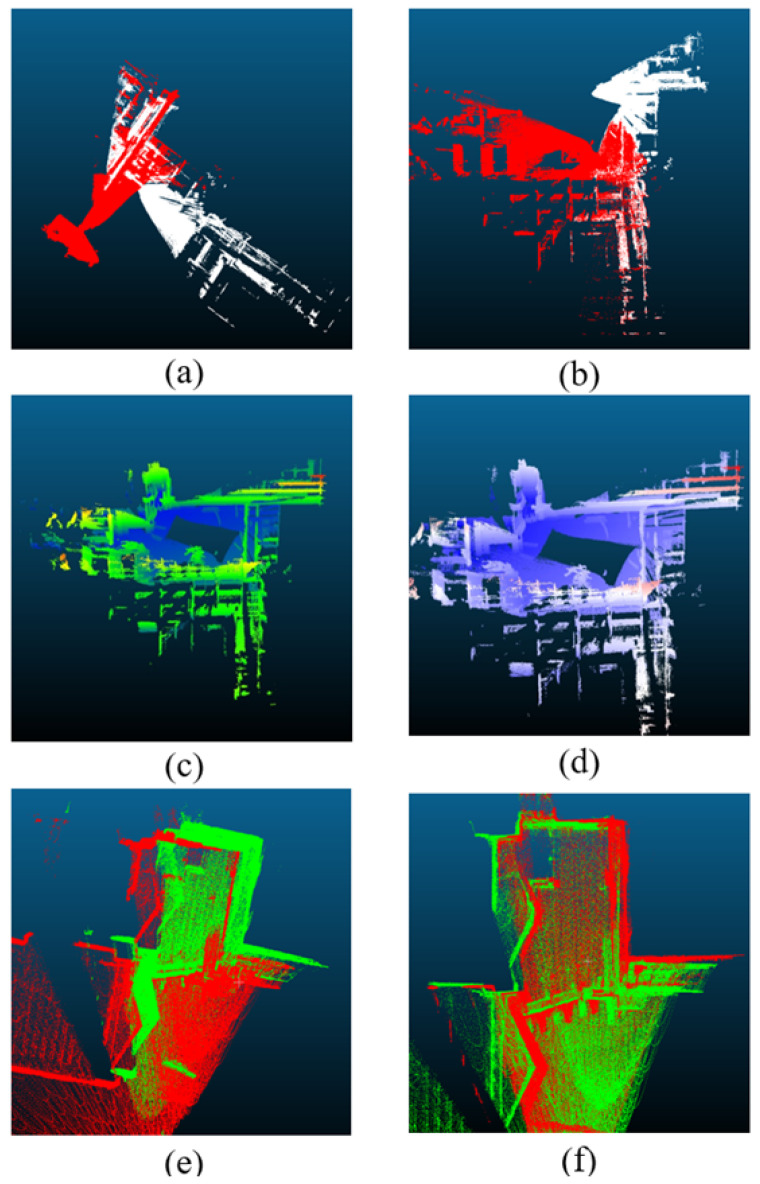
Segmented optimization. Colors stand for different segment.(**a**,**b**) Alignment between two adjacent segments, where red and white point clouds represent consecutive segments. (**c**) Global point cloud map obtained from initial SLAM-based stitching. (**d**) Optimized map after segment-wise registration. (**e**,**f**) Zoomed-in view of the closed-loop area before and after optimization, showing significantly improved alignment.

In the NDT + PL-ICP algorithm, only the overlapping areas between adjacent segments are registered to obtain the transformation relationship between them. This point cloud data is then used to implement an optimized segment-wise registration approach based on NDT+ICP. The results are shown in Figure 5. Figure 5a,b illustrate the registration effects between two adjacent segments, where the red and white point clouds represent successive segments. These images demonstrate strong alignment between adjacent segments. Figure 5c shows the complete point cloud obtained after SLAM processing, while Figure 5d displays the point cloud after segment-wise registration optimization. Comparing Figure 5c,d, a significant accuracy improvement is evident in the closed-loop sections.

For a closer look at the optimization effects in the closed-loop area, Figure 5e,f focus specifically on this region. Figure 5e shows the closed-loop point cloud resulting from the initial SLAM-based stitching, while Figure 5f illustrates the closed-loop point cloud after segment-wise registration optimization, where the white and red point clouds correspond to the starting and ending timestamps, respectively. The comparison between Figure 5e,f clearly highlights the substantial improvement in alignment precision achieved through this optimization, particularly in the closed-loop area.

To validate the optimization effect of the algorithm on SLAM, we manually selected corresponding feature points of the same landmarks from the point clouds at the initial and final timestamps, before and after optimization, and analyzed the errors of these matching feature points. The specific values are shown in Table 1. As can be seen from the table, the distance errors of the corresponding feature points are reduced after optimization, which confirms the improvement in accuracy achieved by the proposed algorithm.

### 4.2. Self-Collected Data

Using the MID-70, a badminton court was scanned, with the entire point cloud segmented into multiple parts during the mobile mapping process. This approach served to validate the accuracy of the NDT + PL-ICP-based mobile mapping segmentation registration and optimization algorithm.

**Figure 6:**
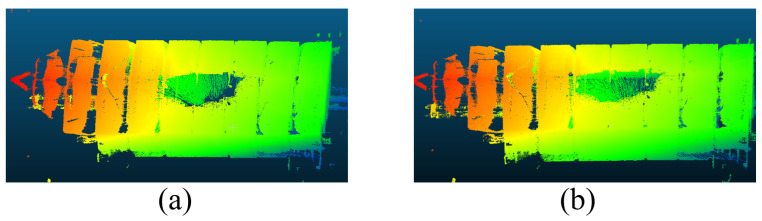
Comparison of complete stitched point cloud before and after optimization. (**a**) is before optimization and (**b**) is after optimization.

**Figure 7:**
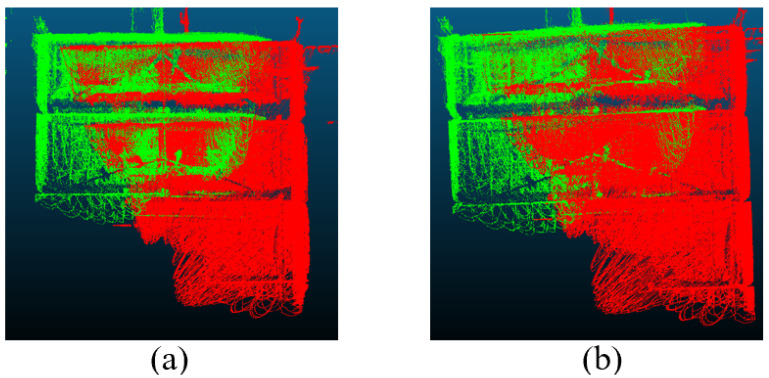
Comparison of point cloud at closed-loop area before and after optimization. (**a**) is before optimization and (**b**) is after optimization.

The point clouds generated using only the SLAM module and those obtained after applying the proposed mobile segmented registration optimization algorithm are presented in Figure 6. As illustrated, the solid-state LiDAR-based SLAM demonstrates good indoor measurement performance with high scanning density. However, Figure 6 alone does not sufficiently demonstrate the magnitude of the SLAM registration error or the effectiveness of the proposed optimization algorithm. To provide a more detailed assessment, a focused comparison of the loop closure region was performed, as shown in Figure 7. In this figure, Figure 7a shows the point cloud in the loop closure area generated by the original SLAM method, while Figure 7b displays the result after applying the segmented registration optimization. The green and red point clouds represent the starting and ending frames, respectively. The color-coded magnified views reveal that the SLAM-stitched point cloud contains notable misalignments, which are significantly reduced after the optimization process.

To further validate the effectiveness of the proposed optimization algorithm in mobile measurement scenarios, we manually selected corresponding feature points from the starting and ending frames in the loop closure region, both before and after optimization. By analyzing the spatial deviations of these matched points, the distance errors were calculated and are presented in Table 2. The results indicate that the point deviations were significantly reduced after optimization, confirming the algorithm’s ability to improve registration accuracy.

In addition, a comparison of average marker errors across different SLAM algorithms is presented in Table 3. The proposed segmented registration algorithm achieves the lowest average error of 0.34 m, outperforming FAST-LIO (0.41 m) and LIO-SAM (0.53 m), thereby further demonstrating its effectiveness in improving SLAM mapping accuracy.

## 5. Conclusions

To address the cumulative errors in frame-by-frame registration encountered by the SLAM algorithm, we conducted experimental measurements to calculate the specific error values. We then proposed a mobile measurement segmented registration optimization algorithm based on NDT + PL-ICP. This method divides the entire point cloud into segments for SLAM stitching and registers overlapping regions of point clouds to enhance the overall stitching accuracy.

Experiments were conducted to verify the practical optimization effect of this algorithm. By comparing the loop closure point clouds from the initial and final frames before and after optimization, and by analyzing the error magnitude based on the coordinates of identical feature points, we confirmed the algorithm’s effectiveness in reducing errors during solid-state LiDAR mobile measurement.

In future work, we plan to explore adaptive segmentation strategies that dynamically adjust segment length and overlap ratio based on scene complexity or motion characteristics. Additionally, we aim to incorporate semantic information and dynamic object modeling into the registration process to further enhance accuracy in complex, unstructured, or dynamic environments. Extending the framework to handle multi-sensor fusion and real-time deployment in large-scale scenarios will also be an important direction for future research.

## Figures and Tables

**Table 1:** Comparison of distance errors for marker points.

ID	1	2	3	4	5	6	7
Error Before Optimization (m)	1.66	1.33	1.7	1.69	1.23	1.19	1.49
Error After Optimization (m)	0.48	0.43	0.31	0.32	0.52	0.62	0.51
Enhancement (m)	1.18	0.9	1.39	1.37	0.71	0.57	0.98

**Table 2:** Comparison of distance errors for marker points.

	Initial Moment Point			Final Moment Point						
	X	Y	Z	X	Y	Z	ΔX	ΔY	ΔZ	ΔS
Before Optimization	16.61	3.03	1.86	15.78	3.16	2.04	0.83	−0.13	−0.18	0.86
	17.14	6.36	1.95	16.11	6.53	2.12	1.02	−0.17	−0.17	1.05
	16.39	3.08	2.88	15.71	3.24	3.19	0.68	−0.16	−0.32	0.77
	22.1	2.12	1.01	21.42	2.56	1.61	0.68	−0.44	−0.6	1.01
After Optimization	16.61	3.03	1.86	16.58	3.24	1.97	0.03	−0.21	−0.11	0.24
	17.14	6.36	1.95	16.99	6.55	1.65	0.15	−0.19	0.3	0.39
	16.39	3.08	2.88	16.12	3.25	2.96	0.27	−0.17	−0.08	0.33
	22.1	2.12	1.01	21.85	2.43	1.05	0.25	−0.31	−0.04	0.4

**Table 3:** Comparison of average marker errors among different algorithms.

	FAST-LIO	LIO-SAM	Ours
error (m)	0.41	0.53	0.34

## Data Availability

The data presented in this study are not publicly available due to privacy and project confidentiality restrictions.
